# Generalized problematic internet use, use of social networks, and appearance schemas in late adolescence

**DOI:** 10.1192/j.eurpsy.2021.1492

**Published:** 2021-08-13

**Authors:** B. Rodrigues Maia, H. Moreira, A. Macedo, A.T. Pereira

**Affiliations:** 1 Faculty Of Philosophy And Social Sciences, Universidade Católica Portuguesa, Braga, Portugal; 2 Center For Research In Neuropsychology And Cognitive Behavioral Intervention, University of Coimbra, Coimbra, Portugal; 3 Institute Of Psychological Medicine, Faculty Of Medicine, University of Coimbra, Coimbra, Portugal; 4 Institute Of Psychological Medicine, Faculty Of Medicine, University of Coimbra, coimbra, Portugal

**Keywords:** social networks, appearance schemas, adolescence, Generalized problematic internet use

## Abstract

**Introduction:**

Research on the association between internet use and concerns about body image is still scarce.

**Objectives:**

To explore the associations between generalized problematic internet use, number of social networks, and appearance schemas.

**Methods:**

216 Portuguese late adolescents (89.3% females), with a mean age of 18.62 years old (SD = .488, range: 18-19) filled in an internet patterns of use questionnaire, the Generalized Problematic Interne Use Scale 2 (GPIUS2) and The Appearance Schemas Inventory-Revised (ASI-R).

**Results:**

A total of 99.6% of the students use social networks. Subjects were divided into three groups (group1: 1-2 social networks; group 2: 3 social networks, and group 3: >3 social networks). There was a statistically difference in Motivational Salience scores (ASI-R) for the three groups [F (2, 503) = 6.0, p = .003]. Post-hoc comparisons indicated that the mean score for group 3 (M = 28.29, SD = 4.95) was significantly different from group 1 (M = 26.69, SD =4.55), and from group 2, (M = 26.87, SD =4.95). Generalized problematic internet subscales (Mood Regulation, Self-Deficient Regulation, and Negative Consequences) and total score were significantly correlated with both dimensions of ASI-R: Self-Evaluation Salience (coefficients varied from r = .31** to r = .47**) and Motivational Salience (from r = .14*, to r = .31**).

**Conclusions:**

Generalized problematic internet use and the number of social networks are associated with adolescent’s cognitive-behavioural investment in one’s own appearance. Study carried out under the strategic project of the Centre for Philosophical and Humanistic Studies (CEFH) UID/FIL/00683/2019, funded by the FCT.

